# The Micro-Organisms of Cancer

**Published:** 1891-08

**Authors:** 


### The Microorganisms of Cancer.

Dr. Schutz(*Centralbl. f. Chir.*, January 24th, 1891), whilst fully recognizing the appearances which have been described by several authors as occurring in cancer, does not, however, regard the bodies in question as living organisms, but thinks it far more probable that they are formed (in part, at least) of red blood corpuscles which, both in carcinoma and sarcoma, find their way freely into the tissues and assume various disguises, so that their origin is not easily determined. Another source of the so-called organisms depends, he asserts, on the alteration of the cells of the various tissues which take upon themselves amœboid movement, and congregate oftentimes in masses; and it is by the alteration of these masses that the so-called sporocysts are formed. So far, he is of opinion we are as far as ever from finding the true organism which produces cancer, even supposing that it exists.—*Brit. Med. Journal.*

